# Autophagy, Inflammation and Innate Immunity in Inflammatory Myopathies

**DOI:** 10.1371/journal.pone.0111490

**Published:** 2014-11-03

**Authors:** Cristina Cappelletti, Barbara Galbardi, Dimos Kapetis, Gaetano Vattemi, Valeria Guglielmi, Paola Tonin, Franco Salerno, Lucia Morandi, Giuliano Tomelleri, Renato Mantegazza, Pia Bernasconi

**Affiliations:** 1 Neurology IV, Neuroimmunology and Neuromuscular Diseases Unit, Fondazione Istituto Neurologico Carlo Besta, Milan, Italy; 2 Section of Clinical Neurology, Department of Neurological and Movement Sciences, University of Verona, Verona, Italy; University of Palermo, Italy

## Abstract

Autophagy has a large range of physiological functions and its dysregulation contributes to several human disorders, including autoinflammatory/autoimmune diseases such as inflammatory myopathies (IIMs). In order to better understand the pathogenetic mechanisms of these muscular disorders, we sought to define the role of autophagic processes and their relation with the innate immune system in the three main subtypes of IIM, specifically sporadic inclusion body myositis (sIBM), polymyositis (PM), dermatomyositis (DM) and juvenile dermatomyositis (JDM). We found that although the mRNA transcript levels of the autophagy-related genes BECN1, ATG5 and FBXO32 were similar in IIM and controls, autophagy activation in all IIM subgroups was suggested by immunoblotting results and confirmed by immunofluorescence. TLR4 and TLR3, two potent inducers of autophagy, were highly increased in IIM, with TLR4 transcripts significantly more expressed in PM and DM than in JDM, sIBM and controls, and TLR3 transcripts highly up-regulated in all IIM subgroups compared to controls. Co-localization between autophagic marker, LC3, and TLR4 and TLR3 was observed not only in sIBM but also in PM, DM and JDM muscle tissues. Furthermore, a highly association with the autophagic processes was observed in all IIM subgroups also for some TLR4 ligands, endogenous and bacterial HSP60, other than the high-mobility group box 1 (HMGB1). These findings indicate that autophagic processes are active not only in sIBM but also in PM, DM and JDM, probably in response to an exogenous or endogenous ‘danger signal’. However, autophagic activation and regulation, and also interaction with the innate immune system, differ in each type of IIM. Better understanding of these differences may lead to new therapies for the different IIM types.

## Introduction

Macroautophagy (or autophagocytosis) is a ubiquitous intracellular catabolic process in which a targeted portion of cytoplasm is enclosed in a double membrane to become an autophagosome, which subsequently fuses with a lysosome. The enclosed cytoplasm is then degraded and the resulting molecules are released and recycled [1]. Amino acids, fatty acids and other cellular components are recycled to provide substrates for macromolecule synthesis and ATP generation [1]. Autophagy seems to be an adaptation to starvation but plays a key role in numerous cellular processes, including differentiation [2], anti-aging [3], tumor suppression/promotion [4], and quality control of intracellular proteins and organelles [5]. During an immune response, autophagy contributes to the degradation of intracellular pathogens, modulates major histocompatibility complex class II (MHC II)-restricted endogenous antigen presentation, is an effector of T-helper 1 (Th1)/Th2 cell polarization, influences B- and T-cell homeostasis and repertoire selection, and assists pattern recognition receptors by delivering pathogen-associated molecular patterns from the cytosol to endosomal Toll-like receptors (TLRs) [6].

Since autophagy has such a large range of physiological functions, it is unsurprising that its dysregulation contributes to several human diseases, including cardiac dysfunction, diabetes, neurodegenerative disorders (Huntington’s, Alzheimer’s and Parkinson’s diseases), cancer, and autoinflammatory/autoimmune diseases [7], including sporadic inclusion body myositis (sIBM) [8]–[10], in which it seems to be responsible for the accumulation of the multiple protein aggregates characteristic of the muscle fibers of this disease [8]. The mechanisms of autophagy dysregulation in sIBM are unknown, and clear descriptions of autophagic processes in other idiopathic inflammatory myopathies (IIMs), specifically polymyositis (PM), dermatomyositis (DM) and juvenile dermatomyositis (JDM) are also lacking. Based on evidence of TLR involvement in autophagy [11]–[16] and findings that these receptors are implicated in the pathogenesis of IIMs [17]–[20], we investigated relations between autophagy and TLRs and other molecules of innate immunity, in PM, DM, JDM, and sIBM.

## Methods

### Muscle biopsies

Muscle samples were obtained from vastus lateralis, deltoid or quadriceps femoris of 43 IIM patients (10 PM, 10 adult DM, 8 JDM and 15 sIBM), all with definite diagnoses based on clinical evaluation and laboratory findings [21], and 10 controls. Open or needle biopsy samples were immediately frozen in liquid nitrogen-pre-cooled isopentane and stored in liquid nitrogen pending assay. Control muscle samples were obtained from patients who underwent biopsy for diagnostic purposes, but had no myopathy. Muscle samples from 4 patients with acid maltase deficiency myopathy served as positive controls for the immunostaining experiments on autophagic molecules, but results are not presented.

### Standard protocol approvals, registrations, and patient consents

The study was approved by the Ethics Committee of the Besta Institute and performed in conformity with institutional review board-approved clinical protocols. Each patient or a parent/guardian provided written informed consent to muscle biopsy for diagnosis and research.

### RNA extraction, cDNA synthesis and quantitative real-time polymerase chain reaction (qPCR)

Total RNA was extracted from 20–30 mg of frozen muscle tissue from each of the 43 IIM patients and 10 controls using TRIzol (Invitrogen, Life Technologies), followed by DNase I treatment (Ambion, Life Technologies). Random-primed cDNA was prepared using the SuperScript VILO cDNA synthesis kit (Invitrogen, Life Technologies) following the manufacturer’s instructions and stored at −20°C pending PCR amplification.

Pre-designed functionally tested assay kits were used for the qPCR analyses: beclin1 (BECN1), Hs00186838_m1; ATG5, Hs00169468_m1; atrogin1 (FBXO32), Hs00369709_m1; TLR4, Hs00152939_m1 (all from Applied Biosystems, Life Technologies). TLR3 transcript expression levels were determined as described elsewhere [17], [22]: TLR3 forward 5-CCTGGTTTGTTAATTGGATTAACGA-3; TLR3 reverse 5-GAGGTGGAGTGTTGCAAAGG-3; TLR3 probe 5-FAM-ACCCATACCAACATCCCTGAGCTGTCAA-3. Relative expression levels of the mRNAs tested were normalized to GAPDH (Hs99999905_m1, Applied Biosystems, Life Technologies) and calculated using 2^−ΔΔCT^ formalism and mean ΔCT value of control samples as calibrator.

### Protein extraction and immunoblotting

Twenty mg of frozen muscle tissue from 3 PM, 3 sIBM, 1 DM, 2 JDM, and 4 controls were lysed in radioimmuneprecipitation assay buffer (50 mmol/L Tris-HCl, pH 8.0, 1% Nonidet P-40, 150 mmol/L NaCl, 0.5% sodium deoxycholate, 0.1% SDS, 1 mmol/L EDTA), freshly supplemented with protease inhibitor cocktail (Sigma-Aldrich) and 1 mmol/L phenylmethanesulfonyl fluoride (Sigma-Aldrich). Protein extracts (40 µg) were separated by NuPAGE Novex 4–12% Bis-Tris protein gels (Invitrogen, Life Technologies), and transferred to a nitrocellulose membrane. After blocking, samples were incubated with antibodies to autophagy-related ubiquitin-like modifier LC3 B (Sigma-Aldrich) and γ-aminobutyric acid type A receptor associated protein (GABARAP) (Abcam), followed by appropriate secondary antibodies. Bound antibodies were visualized by enhanced chemiluminescence with ECL Plus Substrate (Pierce, Thermo Fisher Scientific).

### Double-labeling immunofluorescence

Six µm-thick cryostat cut muscle sections were mounted on polylysine-coated glass slides (Bio-Optica), air-dried for 30 minutes and stored at −80°C pending use. Sections (4 JDM, 4 DM, 4 PM, 4 sIBM, 4 maltase deficiency patients, and 4 controls) were fixed for 10 minutes with 4% paraformaldehyde, permeabilized for 5 minutes with ice cold methyl alcohol, and incubated with 10% normal goat serum diluted in PBS for 30 minutes at room temperature to block non-specific binding. The slides were then incubated overnight at 4°C with various combinations of two primary antibodies (Table S1), washed 3 times with PBS, and incubated with two secondary antibodies (Cy2-conjugated goat anti-mouse immunoglobulin G and Cy3-conjugated goat anti-rabbit immunoglobulin G) (both from Jackson Immunoresearch Laboratories) for 1 h at room temperature. The slides were mounted with FluorSave reagent (Merck Calbiochem), sealed and dried 1 h at room temperature. Images were captured with a Nikon Eclipse TE2000-E confocal laser-scanning microscope and analyzed with EZ-C1 3.70 imaging software (Nikon).

### Immunohistochemistry

Acetone-fixed 6 µm-thick frozen sections from 4 JDM, 4 DM, 4 PM, 4 sIBM, and 4 controls were analyzed for bacterial and human HSP60 protein using anti-bacterial and anti-human HSP60 mouse monoclonal antibodies (both from Alexis Biochemicals). Antibodies were applied for 2 h at room temperature in a humid chamber, followed by 60-min incubation with secondary antibody (DakoCytomation EnVision plus System Labelled Polymer-HRP Anti-Mouse, Dako). Sections were mounted with Bio Mount medium (Bio-Optica) and observed under a Zeiss microscope. As negative controls, sections were incubated with isotype-specific non-immune IgGs (Dako). Single-positive cells were counted on 5 adjacent fields per section at x20 magnification and the results reported as mean percentage and standard deviation (SD).

### Statistical analysis

The Kruskal-Wallis non-parametric test, with Bonferroni post-hoc test, was used to assess differences in mRNA levels in disease groups and controls, since they were not normally distributed (Shapiro-Wilk test *P*<0.05 for each transcript). Differences were considered significant at *P*<0.05. The analyses were performed with R statistical software (version 3.0.2.) (www.r-statistics.org).

## Results

### Transcripts associated with autophagy and innate immunity

**Figure 1:**
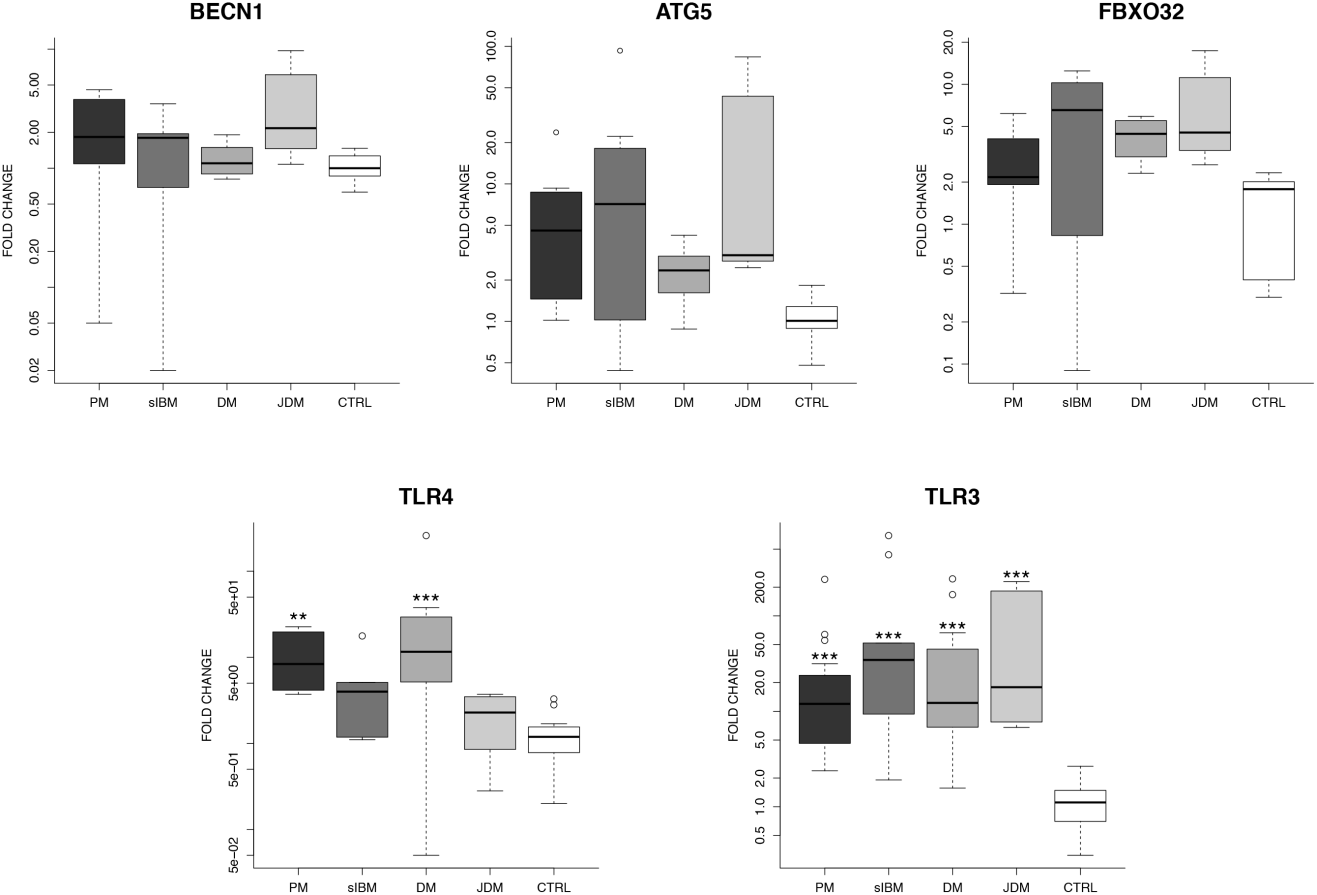
Boxplots of relative gene expression values for the autophagy-related genes and TLRs investigated. Dark horizontal lines represent means, with the box representing the 25th and 75th percentiles, the whiskers the 5th and 95th percentiles, and the dots the outliers. mRNA levels of the autophagy-related genes BECN1, ATG5, and FBXO32 do not differ significantly between groups (IIM and control). By contrast, TLR4 and TLR3 transcripts are upregulated in all IIM subgroups compared to controls; for TLR4, highest expression levels occur in PM and DM muscle (**P<0.01 and ***P<0.001, respectively). PM, polymyositis; sIBM, sporadic inclusion body myositis; DM, dermatomyositis; JDM, juvenile dermatomyositis; CTRL, controls.

The results of quantitative real-time PCR for PM, sIBM, DM, JDM, and controls are shown in Figure 1. mRNA levels of beclin1, mammalian ortholog of yeast Atg6 responsible for phagophore development [1], [23] were similar in IIM and controls. Transcript levels of ATG5, the autophagy-related E3 ubiquitin ligase, necessary for autophagosome elongation [1], [23], and autophagy-related atrogin1 (FBXO32), and involved in skeletal muscle atrophy [24], appeared greater in all IIM groups than controls, but differences were not significant. Consistent with recent findings [17]–[20], TLR4 transcripts were significantly upregulated in PM (*P*<0.01) and DM (*P*<0.001) compared to controls, but not in sIBM or JDM. TLR3 was strongly upregulated in all IIM groups compared to controls (*P*<0.001 for each IIM).

### Autophagy markers in IIM muscles

LC3B and GABARAP proteins were determined by immunoblot of muscle extracts from 3 PM, 3 sIBM, 1 DM, 2 JDM patients, and 4 controls. Cytosolic LC3B-I was present in all samples analyzed. Phosphatidylethanolamine-conjugated LC3B-II was also present in all samples but only weakly present in the 2 JDM homogenates. GABARAP appeared to be more expressed in IIM homogenates than controls (Figure S1), suggesting activation of autophagic processes in all IIM groups.

### TLR4 and TLR3 in IIM muscle

**Figure 2:**
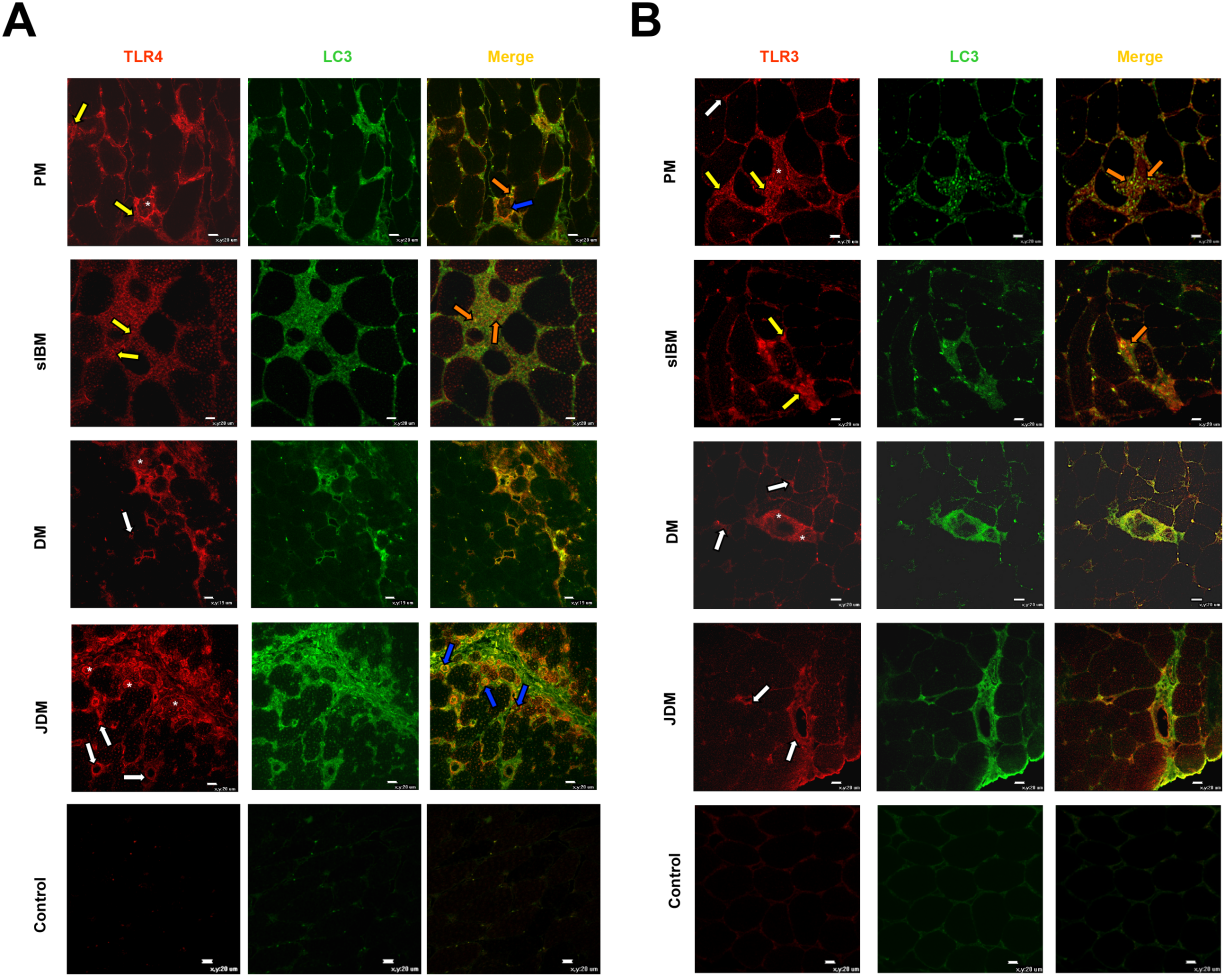
Representative micrographs illustrating localization of TLR4 (A) and TLR3 (B) in IIM and control muscles. (**A**) TLR4 is expressed in association with infiltrating cells in endomysial and perimysial spaces, particularly in PM and sIBM (yellow arrows), on capillaries and large blood vessels, mainly in DM and JDM (white arrows), and with the sarcolemma and sarcoplasm of some muscle fibers. TLR4 positivity is also present on some myofibers in the areas of perifascicular atrophy in DM, mainly in JDM (asterisks). (**B**) TLR3 is present in association with muscle infiltrating cells (yellow arrows), some muscle fibers, particularly atrophic myofibers (asterisks), and vascular endothelial cells (white arrows) in IIM muscle. Weak positivity is also present in control muscle. LC3+ autophagosomes are associated with TLR4+ myofibers (blue arrows) (**A**), and with TLR4+ and TLR3+ inflammatory infiltrates (orange arrows) (**B**). Original magnification x40; bar: 20 µm.

TLR4 immunostaining was weak or absent in control muscles, but clearly evident in all 16 IIM muscle samples examined (Figure 2A). TLR4 was associated with endomysial infiltrating mononuclear cells, mainly in PM and sIBM, and with capillaries and large blood vessels, mainly in DM and JDM. TLR4 was present in the sarcolemma and sarcoplasm of some muscle fibers of all 16 IIMs. In DM and JDM muscle, TLR4 expression was prominent in areas of perifascicular atrophy (Figure 2A). Consistent with previous observations [17], TLR3 positivity was rare or absent in control muscles (Figure 2B), but in all IIM muscles investigated was present in association with infiltrating cells (most contacting muscle fibers), some myofibers, and vascular endothelial cells, particularly in DM and JDM.

### Co-localization of autophagic marker LC3 with TLR4 and TLR3

In PM, and to a similar extent sIBM, TLR4 and LC3 co-localized in muscle infiltrating cells and some myofibers, especially those surrounded by inflammatory infiltrates (Figure 2A). In DM and JDM, LC3 was present in association with most TLR4-positive myofibers and also in association with capillaries and large blood vessels (Figure 2A). TLR3 and LC3 co-localized on infiltrating cells (most contacting muscle fibers) and muscle fibers in all IIM muscle investigated. In DM and JDM, TLR3 and LC3 also co-localized on capillaries and large blood vessels (Figure 2B). In control muscles, TLR4, TLR3, and LC3 staining was weak or absent (Figure 2B).

### Alarmins and autophagy marker LC3 in IIM muscle

**Figure 3:**
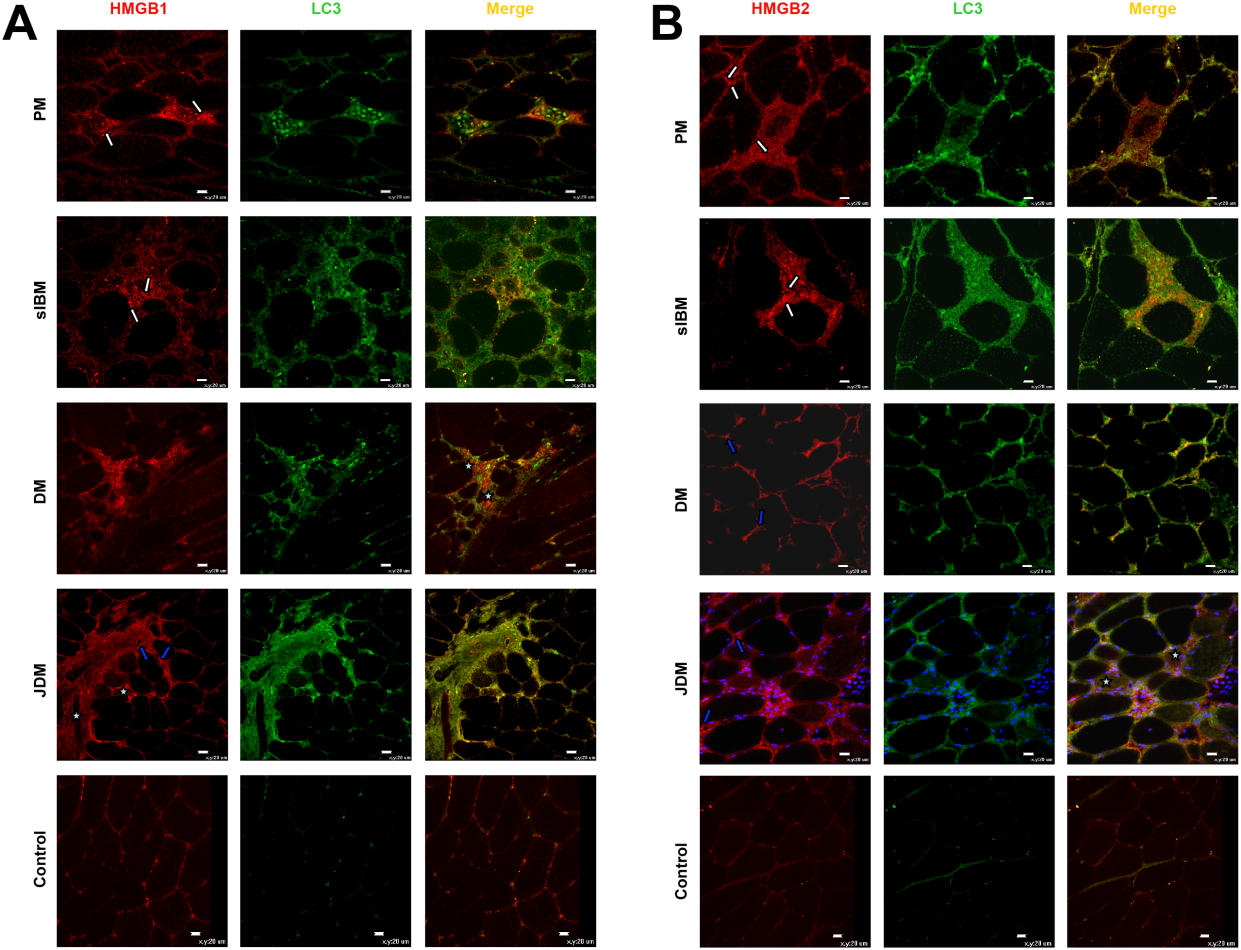
Representative micrographs showing immunolocalization of alarmins (A) HMGB1 and (B) HMGB2 in IIM and controls. Both HMGB1 and HMGB2 are highly expressed in all IIM samples, mainly in association with immune infiltrates (white arrows), blood vessels (blue arrows), nuclei of myofibers, and occasionally with muscle fiber cytoplasm (white asterisks). Both HMGB1 and 2 co-localize with LC3 in association with muscle infiltrating cells and myofibers (white asterisks). In control muscle LC3 and HMGB1 and HMGB2 staining is weak or absent. Original magnification x40; bar: 20 µm.

High-mobility group box 1 (HMGB1) protein, a nonhistone nuclear protein (and alarmin), is present in the sarcoplasm (i.e. outside the nucleus) of muscle fibers without inflammatory infiltrates from early stage IIM patients [25], [26]. We examined the expression of HMGB1 and HMGB2 in the muscle of 16 IIM patients, all of whom were untreated and whose biopsy was taken at an advanced stage when mononuclear cell infiltration was prominent. In all 16 IIM muscles, HMGB1 and 2 were present in association with immune cell infiltrates and muscle fibers, as well as in nuclei (Figure 3). In DM and JDM, HMGB1 and 2 were also present in association with blood vessels, and perifascicular extracellular matrix. Confocal analysis showed that LC3 co-localized with both HMGB1and HMGB2 in cell infiltrates, capillaries, extracellular matrix and myofibers (Figure 3). In control muscles, HMGB1 and HMGB2 staining was weak and present only in nuclei, and LC3 staining was weak or absent (Figure 3).

### Endogenous and bacterial HSP60 in IIM

**Figure 4:**
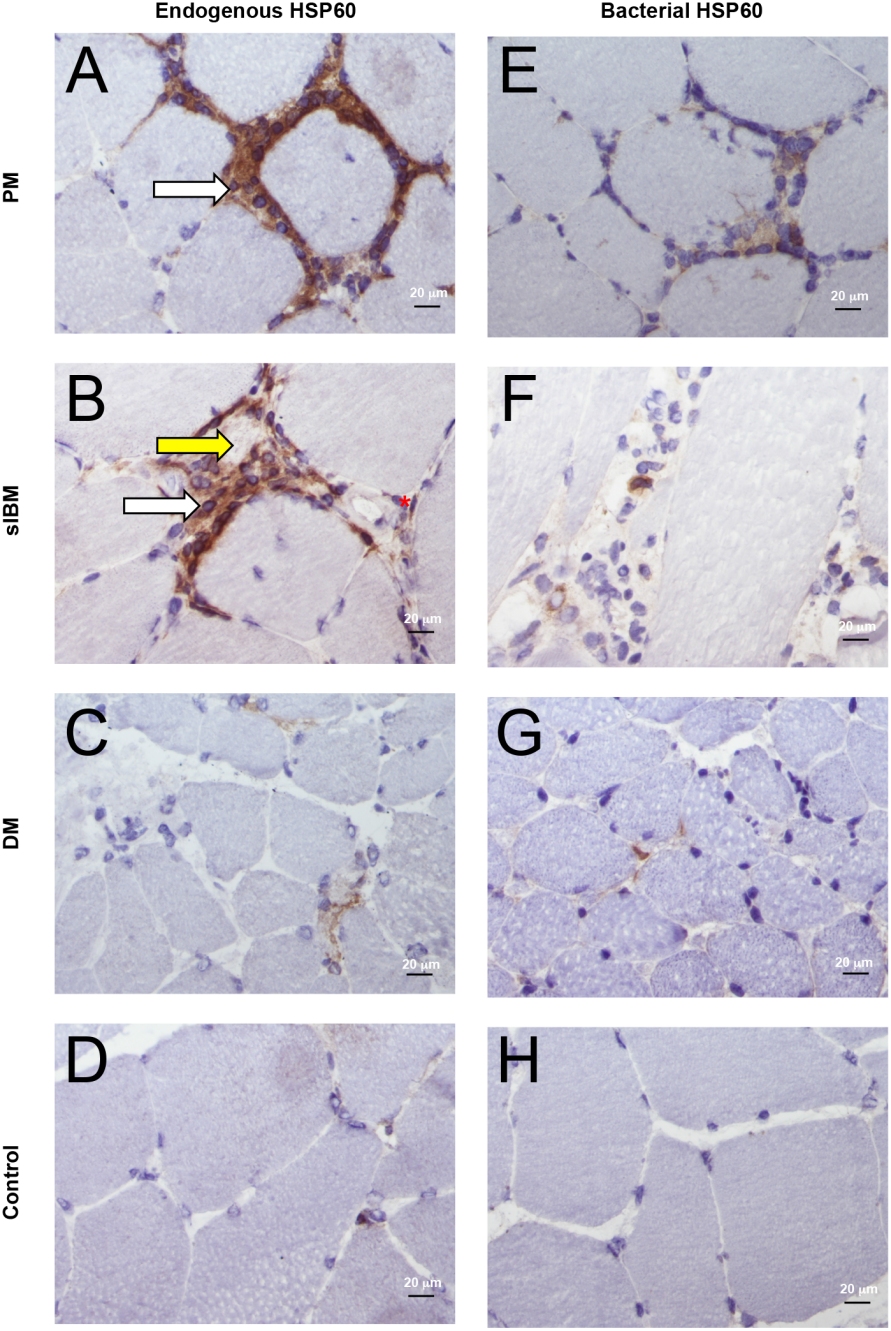
Endogenous and bacterial HSP60 in IIM and control muscles. Endogenous HSP60 is highly expressed in all IIM (**A–C**), mainly in association with inflammatory infiltrates (white arrows), vascular endothelial cells (red asterisk), myofibers surrounded or invaded by immune infiltrates (yellow arrow). In control muscles only occasional capillaries were positive for endogenous HSP60 (**D**). Bacterial HSP60 was occasionally observed associated with muscle infiltrates in all IIM muscles (**E–G**), particularly PM (**E**) and sIBM (**F**), but not controls (**H**). Original magnification x40; bar: 20 µm.

Endogenous HSP60 was present in all 16 IIM muscles examined in association with infiltrating cells, muscle fibers surrounded or invaded by inflammatory infiltrates, capillaries, and blood vessels (Figure 4A–C). In control muscle, endogenous HSP60 was present only occasionally on capillaries (Figure 4D). Bacterial HSP60 was found associated with 22.00% (SD 0.03%), 19.56% (SD 0.07%), 5.10% (SD 1.20%) and 2.80 (SD 1.60%) of infiltrating cells, respectively, in PM, sIBM, DM and JDM (Figure 4E–G); but was not found in control muscles (Figure 4H). Thus both endogenous and bacterial HSP60 were upregulated in IIM. The finding of bacterial HSP60 suggests the presence of a bacterial infection in PM and sIBM muscles.

### Colocalization of endogenous and bacterial HSP60 cells with autophagosome marker LC3

**Figure 5:**
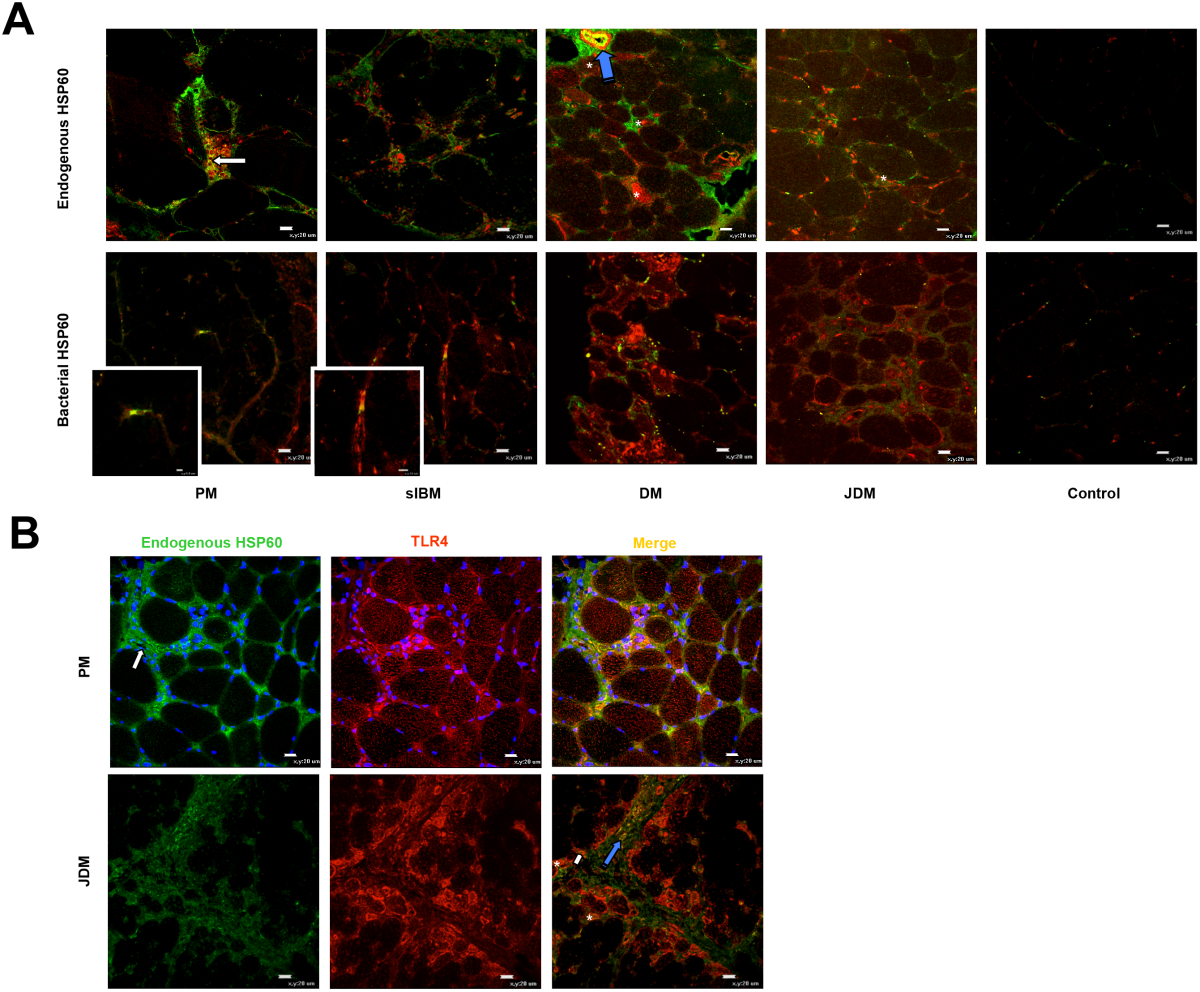
Interaction among HSP60, LC3 and TLR4 in IIM and control muscles. (**A**) LC3-positive autophagosomes (red) are present in infiltrating cells (white arrow), within muscle fibers (asterisks), and blood vessels (blue arrows) (**A**, top panel). Vesicles positive for endogenous HSP60 (green) and LC3 (red) are present mainly in association with infiltrates and capillaries (**A**, top panel). Cells positive for bacterial HSP60 (green) in IIM muscles are also immunopositive for LC3 (red) (**A**, top panel). (**B**) Endogenous HSP60 (green) co-expresses with TLR4 (red) on some muscle fibers (asterisks), blood vessels (blue arrows) and in extracellular matrix (yellow arrows). Original magnification x40; bar: 20 µm. Original magnification of the insets x120; bars: 5 µm (PM) and 10 µm (sIBM).

Since HSP60 is involved in macroautophagy [27], [28], we investigated whether endogenous HSP60 co-localized with LC3 in IIM. We investigated 4 PM, 4 sIBM, 4 DM, 4 JDM and 4 control muscles. We found that endogenous HSP60 co-localized with LC3. In PM and sIBM it co-localized with LC3 mainly in infiltrating cells, whereas in DM and JDM the two proteins co-localized on capillaries, large blood vessels, extracellular matrix and occasionally muscle fibers (Figure 5A). In all 16 IIM muscles investigated, bacterial HSP60-positive cells were also positive for LC3 (Figure 5A and insets), suggesting that autophagy may be involved in clearance of bacterial infection in IIM muscles.

### TLR4 and endogenous HSP60 in IIM muscle

Since TLR4 expression has been shown to be involved in endogenous HSP60-mediated up-regulation of MHC class II and antigen presenting cell accessories [29], we investigated co-localization of TLR4 and endogenous HSP60 in IIM muscle. We found that in all IIM muscles analyzed these proteins co-localized on muscle cell infiltrates, small and large blood vessels, extracellular matrix and some muscle fibers (Figure 5B). In JDM the co-localization was particularly evident on atrophic muscle fibers in perifascicular areas (Figure 5B).

### Autophagy and HLA-DR (MHC class II) in IIM muscle

**Figure 6:**
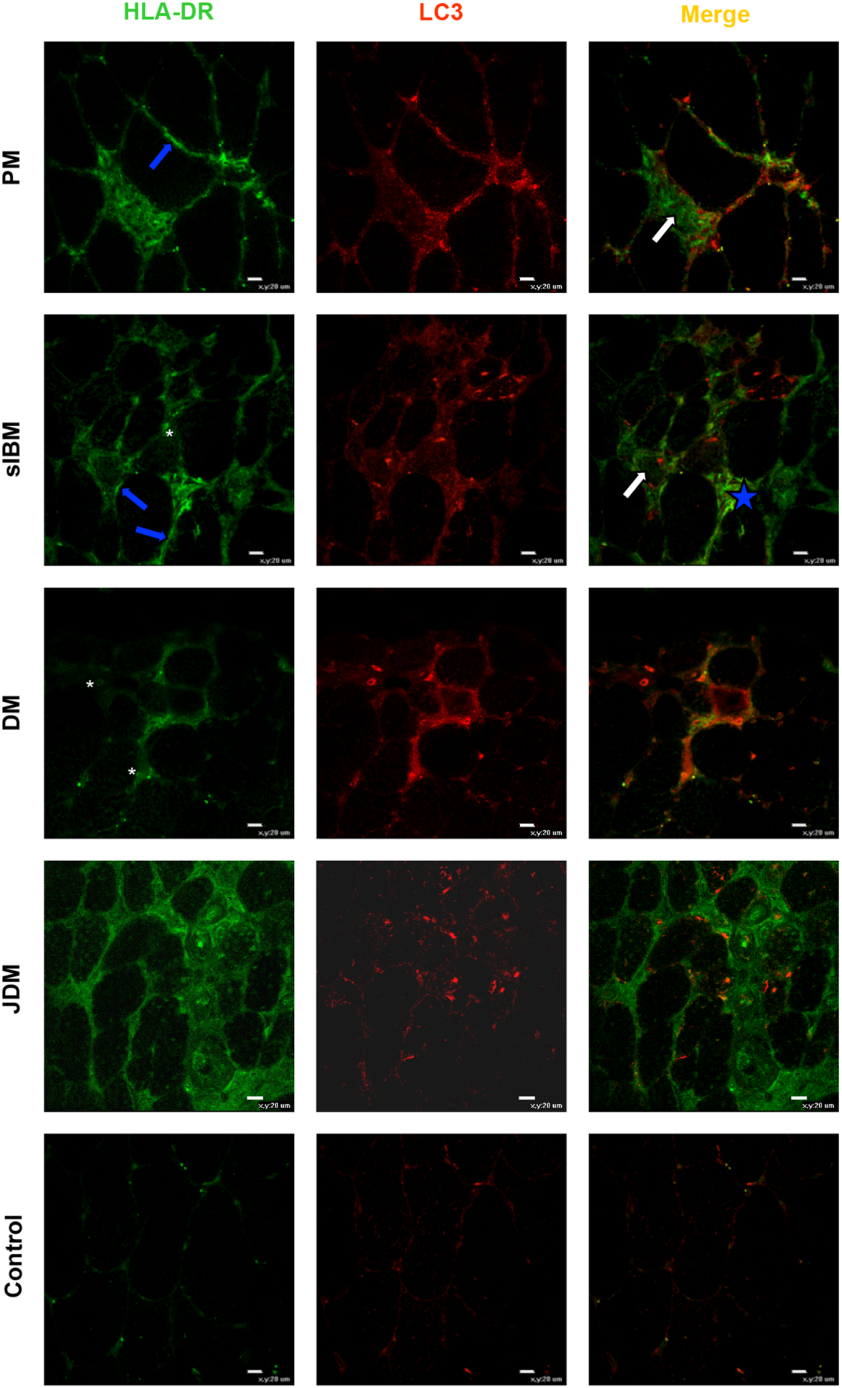
Co-localization of HLA-DR (MHC class II cell surface receptor) with LC3 in IIM and controls. HLA-DR positivity is present on the sarcolemma of some muscle fibers (blue arrows), particularly those close to inflammatory infiltrates in PM and sIBM, and in association with vascular endothelial cells, particularly in DM and JDM (asterisks). HLA-DR-positive myofibers contain LC3-positive vesicles (white arrows) or are close to LC3-positive infiltrating cells (blue star). In control muscles, both positivity for HLA-DR and for LC3 is weak or absent. Original magnification x40; bar: 20 µm.

Recent evidence indicates that autophagy is involved in the generation and sorting of peptides for antigen presentation by MHC class II molecules to T lymphocytes [30]–[33]. Keller and colleagues [33] observed in sIBM muscles that 20% of myofibers contacting CD4+ and CD8+ immune cells were positive for LC3 and MHC class II molecules. In the present study we found that some myofibers positive for HLA-DR also contained LC3-positive structures in PM and sIBM muscle (Figure 6), whereas in DM and particularly JDM co-localization of HLA-DR with LC3 was much less frequent (Figure 6). In control muscles, both HLA-DR and LC3 were weakly expressed or not detected (Figure 6).

### Autophagy and regeneration/degeneration in IIM muscle

In view of the importance of autophagy in preserving muscle mass, maintaining myofiber integrity [34], and the regeneration of endothelial cells [35], we investigated whether LC3 was associated with degenerating or regenerating muscle fibers in 16 IIM muscle biopsies. We found that most muscle cells positive for developmental myosin heavy chain (marker of regeneration) were also rich in LC3-positive autophagosomes (PM: 70.27% SD 0.26%; sIBM: 91.67% SD 0.14%, DM and JDM: 100 SD 0%), whereas the degeneration marker C5b9 rarely co-localized with LC3 and was observed only in IIM muscle fibers invaded by infiltrating cells (not shown).

## Discussion

Impaired autophagosome maturation with consequent accumulation of multiprotein aggregates is considered a key factor in the myofiber degeneration and muscle weakness characteristic of sIBM [8]–[10]. However, it is unclear whether the autophagic pathway is also altered in other IIMs [36]. We therefore investigated markers of autophagy in muscles from PM, DM and JDM patients in comparison to sIBM and control muscle.

We found no differences between IIM and control muscles for transcript levels of the autophagy-specific genes BECN1 and ATG5, whose proteins are necessary for phagophore development and autophagosome elongation, respectively [23]; or for transcripts of atrogin 1 (FBXO32), a muscle-specific atrophy-related ubiquitin ligase also associated with autophagy [24].

Nevertheless it is known that certain forms of macroautophagy are induced independent of BECN1- [37], and also of ATG5 [38], [39]. So we pursued our investigation of autophagy in IIMs by examining the expression of the proteins LC3 and GABARAP – both important markers of autophagy [38]. We found that cytosolic LC3B-I and phosphatidylethanolamine conjugated LC3B-II were expressed in all IIM muscles analyzed, but they were also expressed in controls, whereas GABARAP protein levels appeared higher in IIMs than controls (quantitation not performed), suggesting that the later stage of the autophagosome maturation is occurring in IIM muscles [38].

A growing body of evidence suggests that TLRs are involved in autophagy [11]–[16]. And lipopolysaccharides (TLR4 ligand) and TLR3 and TLR7 agonists have been shown to be potent inducers of autophagy in macrophages [14], [15]. Since TLR3 and TLR4 are also implicated in IIM pathogenesis [17]–[20], we also looked for associations between TLR3 and TLR4 and autophagy in IIM. We found that TLR3 transcripts were significantly upregulated in all IIM samples compared to controls. We also found, by immunofluorescence that TLR3 protein was abundantly expressed in IIM muscles, but was rarely expressed or undetected in control muscle.

TLR4 transcripts were also significantly upregulated in PM and DM, but not in JDM or sIBM – as also reported by Brunn et al. [20]. Similarly we found TLR4 protein was highly expressed in all IIMs by immunofluorescence but was practically absent from control muscle.

We next performed confocal microscopy and found that TLR4 and TLR3 co-localized with the autophagic marker LC3 in all IIM muscle analyzed. In PM and sIBM, this co-localization was observed mostly in association with muscle infiltrating cells and some myofibers, especially those surrounded by inflammatory infiltrates, whereas in DM and especially in JDM, LC3 was often found in TLR4-positive or TLR3-positive myofibers and blood vessels.

These co-localization data suggest co-activation of TLR-mediated innate immunity and autophagy in IIM muscles. Such co-activation suggests a host response to inflammation (possibly due to microbial infection) in PM and sIBM, or stress (due to disease-related capillary depletion leading to hypoxia, ROS accumulation and nutrient deprivation) in DM and JDM. Co-activation of TLR-mediated innate immunity and autophagy is also supported by our finding that HMGB1 and HSP60, (TLR4 ligands) were highly expressed in IIM muscles but immunostaining was weak or absent in controls.

Local HMGB1 release from muscle fibers or other cells is thought to be necessary for inducing muscle dysfunction in patients with PM or DM [26]. HMGB1 release also serves as a trigger for autophagy [40]. We observed co-localization of HMGB1 with LC3 in muscle-infiltrating cells in PM and sIBM, suggesting HMGB1 involvement in the inflammatory process. In particular, and as previously suggested [41], LC3-positive (autophagic) vesicles might have a role in the transport of HMGB1 from the immune cell nucleus to the extracellular space. On the other hand, the association of HMGB1 with LC3 on capillaries and large blood vessels in DM and JDM suggests that HMGB1 may be involved in promoting angiogenesis and perfusion recovery, as recently demonstrated in cultured dermal microvascular endothelial cells and in the hind limb of a mouse ischemia model [42]. These findings further support the idea that in PM and sIBM the main players of the pathogenetic process are muscle-infiltrating mononuclear cells, whereas in DM, involvement of the microvasculature is crucial for the onset or progress of the disease.

HSP60, whose effects are mediated by TLR4 signaling, is one of the most important ‘danger-associated’ molecules produced by stressed tissue [43], [44]. It has been demonstrated that reactivity to HSP60 is involved in the disease process in JDM [45]. In the present study we found that endogenous HSP60 was highly expressed not only in JDM but also in all the other IIMs. HSP60 was present in connective tissue, associated with infiltrates, with muscle fibers surrounded or invaded by infiltrates, mostly in PM and sIBM, and with capillaries and large blood vessels, mainly in DM and JDM. Like HMGB1, HSP60 has been suggested to be involved in the induction of autophagy [27]. Our finding that HSP60 and LC3 co-localized in all 16 IIM muscles examined, is consisted with HSP60 involvement in autophagy. However, in PM and sIBM, HSP60 co-localized with LC3 mainly in association with muscle infiltrating cells, whereas in DM and JDM co-localization was mainly on capillaries, large blood vessels and connective tissue, and occasionally in association with muscle fibers. Thus, autophagic processes appear to differ according to myositis type: in PM and sIBM inflammation or pathogen infection (as suggested by the presence of infiltrating cells positive for bacterial HSP60) could be the main triggering factor for autophagy; whereas in DM and JDM, ischemia or reperfusion injury combined with the presence of oxidative stress could activate autophagy, as recently proposed for myocardial tissue [46]. Detection of bacterial infections has been well documented in PM and DM [47]–[49], whereas, to our knowledge, our study is the first reporting the presence of a bacterial antigen in sIBM muscle.

Recent evidence also indicates that autophagy is involved in the generation and sorting of peptides for antigen presentation by MHC class II molecules to T lymphocytes [30]–[32]. For sIBM it has been reported that 20% of myofibers contacting CD4+ and CD8+ immune cells are positive for both LC3 and MHC class II molecules [33]. We found, in all IIMs investigated, that HLA-DR and LC3 co-localized on the sarcolemma, particularly in areas in close contact with cell infiltrates, as well as in the sarcoplasm of some myofibers, that in all cases were surrounded by cellular infiltrates and capillaries. These findings reinforce the hypothesis that muscle cells contribute to maintaining an inflamed microenvironment in IIMs. We suggest that this is due to autophagy-dependent antigen presentation.

Finally, the well-established role of autophagy in the preservation of muscle mass and myofiber integrity [34], along with the relation between TLR3 and autophagy observed in the present study, and the TLR3 involvement in DM and PM muscle regeneration we previously showed [17], prompted us to investigate the links between autophagy and degeneration and regeneration in IIM muscle. By confocal microscopy, we found that high percentages of muscle cells positive for developmental myosin heavy chain (marker of regeneration) were also rich in LC3-positive autophagosome vesicles. By contrast, the degeneration marker C5b9 rarely colocalized with LC3 in muscle fibers surrounded or invaded by immune cells. These findings suggest autophagy may be involved in limiting muscle damage and in promoting repair in the skeletal muscle of IIM patients, rather than in skeletal muscle degradation.

To conclude, our results show that autophagy is involved in the pathogenesis of all IIMs. However autophagic activation and regulation, and also interaction with the innate immune system, differ in each type of IIM. Better understanding of these differences may be expected to lead to new and distinct therapies for the different IIM types.

## Conclusions

Autophagy plays a key role in IIM pathogenesis but its activation, regulation and interaction with the innate immune system differ depending on IIM subtype.

## Supporting Information

Figure S1 **Immunoblots of muscle lysates from IIM patients and controls revealing the presence of LC3B-I, LC3B-II and GABARAP in all samples analysed.** α-tubulin was loading control.
(TIF)

Table S1 **List of antibodies used in this study.**
(XLS)
