# Supplementary figures and images for: Autophagy, Inflammation and Innate Immunity in Inflammatory Myopathies
Source: PLoS One. 2014 Nov 3;9(11):e111490. doi: 10.1371/journal.pone.0111490 (PMC4218755; doi:10.1371/journal.pone.0111490)

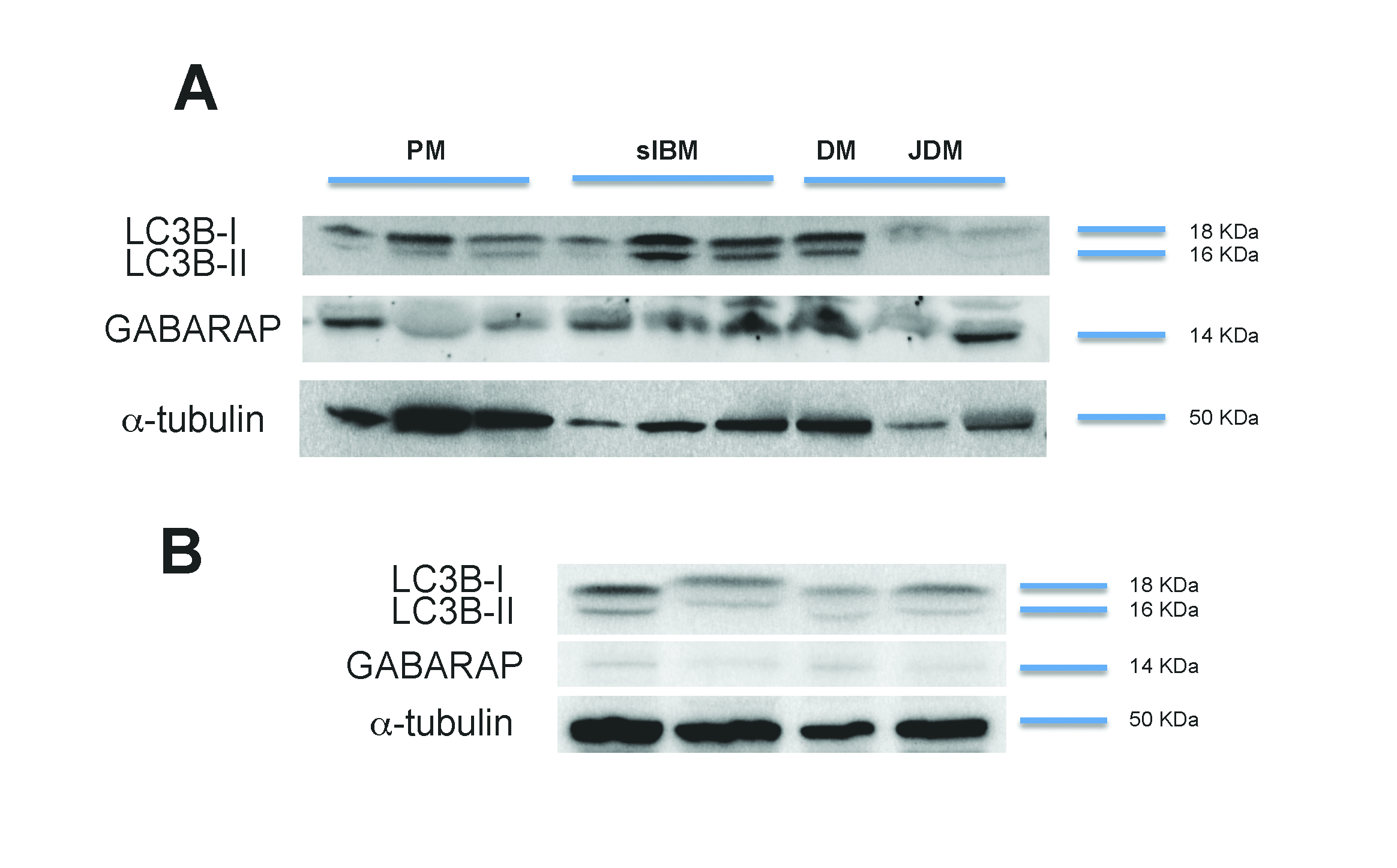

Supplement: Figure S1 — Immunoblots of muscle lysates from IIM patients and controls revealing the presence of LC3B-I, LC3B-II and GABARAP in all samples analysed. α-tubulin was loading control. (TIF) [file pone.0111490.s001.tif]
